# A Rapid Assessment of the Availability and Use of Obstetric Care in Nigerian Healthcare Facilities

**DOI:** 10.1371/journal.pone.0039555

**Published:** 2012-06-22

**Authors:** Daniel O. Erim, Usman M. Kolapo, Stephen C. Resch

**Affiliations:** 1 Center for Health Decision Science, Department of Health Policy and Management, Harvard School of Public Health, Boston, Massachusetts, United States of America; 2 Indepth Precision Consult, Wuse Zone 4, Abuja, Nigeria; 3 Harvard University Center for Geographic Analysis, Cambridge, Massachusetts, United States of America; Tehran University of Medical Sciences, Iran (Islamic Republic of)

## Abstract

**Background:**

As part of efforts to reduce maternal deaths in Nigeria, pregnant women are being encouraged to give birth in healthcare facilities. However, little is known about whether or not available healthcare facilities can cope with an increasing demand for obstetric care. We thus carried out this survey as a rapid and tactical assessment of facility quality. We visited 121 healthcare facilities, and used the opportunity to interview over 700 women seeking care at these facilities.

**Findings:**

Most of the primary healthcare facilities we visited were unable to provide all basic Emergency Obstetric Care (bEmOC) services. In general, they lack clinical staff needed to dispense maternal and neonatal care services, ambulances and uninterrupted electricity supply whenever there were obstetric emergencies. Secondary healthcare facilities fared better, but, like their primary counterparts, lack neonatal care infrastructure. Among patients, most lived within 30 minutes of the visited facilities and still reported some difficulty getting there. Of those who had had two or more childbirths, the conditional probability of a delivery occurring in a healthcare facility was 0.91 if the previous delivery occurred in a healthcare facility, and 0.24 if it occurred at home. The crude risk of an adverse neonatal outcome did not significantly vary by delivery site or birth attendant, and the occurrence of such an outcome during an in-facility delivery may influence the mother to have her next delivery outside. Such an outcome during a home delivery may not prompt a subsequent in-facility delivery.

**Conclusions:**

In conclusion, reducing maternal deaths in Nigeria will require attention to both increasing the number of facilities with high-quality EmOC capability and also assuring Nigerian women have access to these facilities regardless of where they live.

## Introduction

The total number maternal deaths worldwide has reportedly dropped by a third over the last decade, yet over one-third of a million women worldwide still die from maternal causes, more so in low and middle-income countries [1]. Nigeria accounts for 1 in 6 maternal deaths globally, with approximately 50,000 maternal deaths occurring each year [1], [2], [3], [4]. The Nigerian Government and its partners are confronting this challenge by providing more contraceptives, skilled birth attendants, subsidized maternal services, and by promoting in-facility deliveries [5], [6], [7], [8], [9]. As more births are occurring in healthcare facilities, there isn’t much data to show if these facilities can cope with an increasing demand for obstetric care. These data are vital when planning scaling-up of intrapartum services such as assisted vaginal delivery, removal of placenta and retained products, providing parenteral antibiotics, oxytocics and anticonvulsants (all of which form basic emergency obstetric care services or basic EmOC), blood transfusion and Caesarean section (constituting comprehensive EmOC services when basic EmOC services are available). A recent study suggests that poor facility quality may undermine efforts to reduce maternal and perinatal morbidity and mortality [10]. Hence we conducted an in-country survey as a rapid and tactical assessment of facility quality in Nigeria, and to provide a baseline against which efforts to scale up intrapartum care may be evaluated.

## Methods

We undertook a cross sectional survey of randomly selected Nigerian healthcare facilities, and a convenient sample of women receiving maternal care at these facilities. While designing this study, Nigeria’s geopolitical structure was taken into consideration. Nigeria has six geopolitical zones, each of which contains five to seven contiguous states. As maternal indices appear to be similar across states within each zone, one state was randomly chosen from each zone, and they are as follows: Kwara (north central), Sokoto (northwest), Gombe (northeast), Ebonyi (southeast), Delta (south south), and Ondo (southwest) [11]. The Ministry of Health in these states then provided a list containing the names and addresses of all healthcare facilities in their respective state. From these lists, 20 facilities per state (10 primary, 6 secondary and 4 tertiary) were randomly selected, and in states with less than 4 tertiary hospitals, additional secondary hospitals were selected as substitutes. Survey interviewers then visited each facility on a randomly chosen workday in May 2011.

We were interested in the availability, quality, and cost of routine and emergency obstetric services, as well as the experiences women have accessing obstetric care, and we created two sets of questionnaires to collect data on these. The first set inquired into facility infrastructure. They survey interviewers administered them to principal officers of the selected healthcare facilities, and responses were checked against facility records. The second set was administered to women aged 15–49 years, who were seeking a maternal service at the facility on the day it was being visited by the survey interviewers. The questionnaires inquired into respondents’ socioeconomic and demographic circumstances, experiences while accessing obstetric care from skilled and unskilled providers, delays in accessing care, obstetric history and future plans for delivery among those who knew they were pregnant. Thus, at each visited facility, a principal officer and 6 or 7 female clients of reproductive age were interviewed after obtaining written consent from them. In all, 120 facilities and 738 women were interviewed with a response rate above 99%. IRB approval was obtained from the Harvard School of Public Health and the National Health Research Ethics Committee of Nigeria (NHREC) while the Nigerian Federal Ministry of Health provided logistic support.

Data from the questionnaires were captured electronically using Census and Survey Processing System (US Census Bureau Washington DC, USA) and triple-checked for consistency, The data was then converted to a Stata® compatible file format using Stat/Transfer 11 (Circle systems, Seattle, USA), and analyzed using StataSE® 10.1 for Macintosh.

**Table 1:** Some measures of facility capacity.

	Ownership			Source of electricity	Public source	Generator	Solar	None
Level of care provided	Public	Private	Total					
Primary healthcare	44	17	61	Primary healthcare centers (n = 61)	25 (41%)	16 (26.2%)	3 (3.3%)	32 (52.6%)
Secondary healthcare	48	3	51	Secondary healthcare centers (n = 51)	46 (90.2%)	38 (74.5%)	4 (7.8%)	4 (7.8%)
Tertiary healthcare	0	0	9	Tertiary healthcare centers (n = 9)	9 (100.0%)	9 (100.0%)	1(11.1%)	-
Total	101	20	121	Public healthcare facilities (n = 101)	67 (66.3%)	49 (45.8%)	8 (7.9%)	33 (32.7%)
				Private healthcare facilities (n = 20)	13 (65.0%)	14 (70.0%)	-	3 (15.0%)

Service provision	All facilities(n = 121)	Primary carefacilities (n = 61)	Data fromsimilar studies	Secondary carefacilities (n = 51)	Data fromsimilar studies	Tertiary carefacilities (n = 9)	Public facilities(n = 101)	Private facilities(n = 20)
Can always provide emergency obstetric care	83 (68.6%)	25 (41.0%)	0% [19]	49 (96.1%)	-	9 (100.0%)	65 (65.7%)	17 (81.0%)
Can administer injectable antibiotics	105 (86.8%)	48 (78.7%)	70% [19]	48 (94.1%)	93% [19]	9 (100.0%)	87 (86.1%)	18 (90.0%)
Can diagnose and treat eclampsia	50 (41.3%)	14 (23.0%)	28% [19]	28 (54.9%)	87% [19]	9 (100.0%)	42 (41.6%)	8 (40.0%)
Can conduct assisted vaginal delivery	37 (30.6%)	6 (9.8%)	11% [19]	22 (43.1%)	39% [19]	9 (100.0%)	31 (30.7%)	7 (30.0%)
Can diagnose and treat severe shock	76 (62.8%)	22 (36.1%)	-	45 (88.2%)	-	9 (100.0%)	63 (62.4%)	13 (65.0%)
Can safely transfuse blood	66 (54.5%)	12 (19.7%)	-	45 (88.2%)	88% [19]	9 (100.0%)	53 (52.5%)	13 (65.0%)
Can carry out caesarian sections	56 (46.3%)	7 (11.5%)	-	40 (78.4%)	85% [19]	9 (100.0%)	47 (46.5%)	9 (45.0%)

Staff availability (at least one of the followingclinicians is constantly available)	All facilities(n = 121)	Primary carefacilities (n = 61)	Data fromsimilar studies	Secondary carefacilities (n = 51)	Data fromsimilar studies	Tertiary carefacilities (n = 9)	Public facilities(n = 101)	Private facilities(n = 20)
A doctor	69 (57.0%)	12 (19.7%)	-	48 (94.1%)	-	9 (100.0%)	57 (56.4%)	12 (60.0%)
A nurse/midwife	85 (70.2%)	29 (47.5%)	-	47 (92.2%)	-	9 (100.0%)	69 (68.3%)	16 (80.0%)
An obstetrician	33 (27.3%)	5(8.2%)	-	19 (37.3%)	-	9 (100.0%)	29 (28.7%)	4 (20.0%)
A pediatrician	21 (17.4%)	3 (4.9%)	-	9 (17.6%)	-	9 (100.0%)	17 (16.8%)	4 (20.0%)
An anesthesiologist	32 (26.4%)	4 (6.6%)	-	19 (37.3%)	-	9 (100.0%)	27 (26.7%)	5 (25.0% d)

Infrastructure	All facilities(n = 121)	Primary carefacilities (n = 61)	Data fromsimilar studies	Secondary carefacilities (n = 51)	Data fromsimilar studies	Tertiary carefacilities (n = 9)	Public facilities(n = 101)	Private facilities(n = 20)
Has an ambulance for transporting pregnantwomen to referral facilities	41 (31.2%)*	3 (4.9%)	-	34 (66.7%)	-	Not applicable	34 (37.0%) ‡	3 (15.0%)
Has at least one labor ward	108 (89.3%)	48 (78.7%)	-	51 (100%)	-	9 (100.0%)	90 (89.1%)	18 (90.0%)
Has a least one delivery room	104 (86.0%)	45 (73.8%)	-	51 (100%)	-	9 (100.0%)	86 (85.1%)	18 (90.0%)
Has at least one functional operating room	67 (55.4%)	10 (16.4%)	-	48 (94.1%)	-	9 (100.0%)	57 (56.4%)	10 (50.0%)
Has a neonatal ward/ICU	38 (31.4%)	6 (9.8%)	-	24 (47.1%)	-	8 (88.9%)	33 (32.7%)	5 (25.0%)
Receives uninterrupted electricity supplywhenever there are obstetric emergencies.	37 (30.6%)	9 (14.8%)	-	19 (37.3%)	-	9 (100.0%)	27 (26.7%)	10 (50.0%)

Primary care facilities are synonymous with primary healthcare facilities. This also applies to secondary and tertiary care facilities. Tertiary care facilities have all the necessary infrastructure and resources to provide optimal emergency obstetric care. However, they are very few relative to primary and/or secondary facilities.

ICU  =  intensive care unit; n  =  sample size.

* n = 112; ‡ n = 92.

## Results

### Facility Quality

All the visited facilities provided some form of antenatal care and/or delivery service. While most of the referral facilities could provide emergency obstetric care on a 24-hourly basis, only 40% of primary healthcare facilities could do this. Additionally, some emergency obstetric services (termed “signal functions” by the WHO [12]) were assessed, and found to be unavailable in most primary healthcare facilities (see Table 1). About half of the primary healthcare facilities we visited always had at least one nurse/midwife on-duty. However, they all lacked doctors, especially specialist obstetricians, pediatricians and anesthesiologists. While three quarter of all primary healthcare facilities had a “Labor ward” and a separate “Delivery room”, most of them lacked neonatal wards/intensive care units (NICU), or guaranteed power supply whenever there were obstetric emergencies. In all respects, secondary healthcare facilities fared better, but, like their primary counterparts, lack neonatal wards and ICUs.

### Respondents’ Characteristics

While almost 4 in 5 respondents were younger than 35 years, over 50% had at least secondary-level education, about 70% were employed, and approximately 90% were married (see Table 2). About 3 in 5 respondents were pregnant, and while over three quarter of them planned to deliver in a healthcare facility, five percent of those who planned to deliver at home wanted a skilled attendant to supervise the delivery (see Table 2). Additionally, more than half of all pregnant respondents planned to pay up to US$20 for delivery services, and over half of the remainder didn’t intend to pay anything.

Over 80% of all respondents spent less than 30 minutes getting to the facility, and almost all spent less than $1 doing so (including the cost of transporting whoever accompanied them). Most of them travelled to the facility on foot or via a commercial motorcycle. Lack of partner’s permission and/or funds were significant challenges to accessing care for over half the respondents.

### Pregnancy Outcomes

Of the 736 women in our sample, 600 had previously been pregnant. These 600 women reported 1,704 pregnancies, and they occurred between 1979 and 2011 (75% occurred between 2000–2011). About 4% of these pregnancies reportedly ended in a stillbirth (vs. 4.2% by Cousens et al [13]), 6.6% ended as a spontaneous abortion (vs. 7.8% by Okonofua et al [14]), while 2.4% were electively terminated (vs. 9–12 per 100 pregnancies by Henshaw et al. [15]). The crude risk of a reported stillbirth did not significantly vary by delivery site (i.e. in-facility vs. at home), or by who supervised the delivery (i.e. skilled vs. unskilled birth attendant; see Table 3). However, the risk of a reported adverse neonatal outcome (excluding low birth weight) was significantly higher in home deliveries (see Table 3). The most commonly reported maternal complication was fever (16%), followed by obstructed labor (4.2%), prolonged vaginal bleeding (3.5%), and eclampsia (1.2%).

**Table 2:** Data on respondent characteristics, delivery plans, transportation to the facility and abortions.

**Total number of respondents = 736**								
**Age**	**N (%)**	**2008 DHS**	**Highest level of formal education**	**N**	**2008 DHS**	**Marital status**	**N (%)**	**2008 DHS**
15 – 19 years	88 (12.0%)	19.4%	None	179 (24.5%)	35.8%	Single	58 (7.9%)	25.2%
20 – 24 years	174 (23.6%)	18.4%	Some primary	89 (12.2%)	6.1%	Married	649 (88.2%)	69.1%
25 – 29 years	181 (24.6%)	18.9%	Finished primary	77 (10.5%)	13.6%	Divorce/separated	19 (2.6%)	1.9%
30 – 34 years	135 (18.3%)	13.9%	Some secondary	100 (13.7%)	18.1%	Widowed	10 (1.4%)	2.3%
35 – 39 years	95 (12.9%)	11.7%	Finished secondary	153 (20.9%)	17.5%			
40 – 44 years	47 (6.4%)	9.1%	More than secondary	134 (18.3%)	8.9%			
45 – 49 years	16 (2.2%)	8.6%						
Mean = 27.8 years								
**Currently pregnant (all)**	**N (%)**		**Currently pregnant (15–19 years)**	**N (%)**		**Employment status**	**N (%)**	**2008 DHS**
Yes	433 (58.8%)		Yes	63 (71.6%)		Unemployed	217 (29.5%)	40.8%
No	290 (39.4%)		No	24 (27.3%)		Employed	519 (70.5%)	59.1%
Don’t know	13 (1.8%)		Don’t know	1 (1.1%)				
**Plans for place of delivery**	**N (%)**		**Plans for who to supervise**	**In facility**	**At home**	**Plans for how much to pay for delivery services**		
At a facility	333 (76.9%)		**the delivery**	**N (%)**	**N (%)**	**Amount**	**N (%)**	
At home	83 (19.2%)		Skilled birth attendant	337 (100%)	5 (4.9%)	<US$ 1.00	13 (3.0%)	
At a TBA's home	10 (2.3%)		Traditional birth attendant	-	74 (73.3%)	US$1.00 – US$19.00	217 (50.1%)	
At church	7 (1.6%)		A female relative	-	3 (3.0%)	US$20.00 – US$39.00	50 (11.5%)	
			No one would assist	-	19 (18.8%)	≥ US$40.00	14 (3.2%)	
						Did not intend to pay	139 (32.1%)	
**Transportation to facility**			**Average cost** **	**Average cost** **				
**Means of transport today** *	**N (%)**	**Ave. duration**	**(US dollars)**	**(Nigerian naira)**		**Most likely reason not to visit this facility when the need arises**		
Commercial motorcycle	330 (44.8%)	23 min	US$0.50	N 78.11		**Reason**	**N (%)**	
Walked	233 (32.2%)	19 min	US$0.00	N 0.00		No permission	297 (40.4%)	
Taxi	77 (10.5%)	36 min	US$0.69	N 107.79		No money for treatment	139 (18.8%)	
Personal automobile	42 (6.1%)	25 min	US$0.24	N 37.49		Waiting time is too long	57 (7.7%)	
Bus	42 (5.7%)	37 min	US$0.72	N 112.48		None	57 (7.7%)	
Bicycle	9 (1.2%)	35 min	US$0.33	N 51.55		No means of transport	50 (6.8%)	
Boat	3 (0.4%)	17 min	US$0.75	N 117.17		Feeling weak/tired	34 (4.6%)	
						The facility is too far	29 (3.9%)	
						If I’m very busy	16 (2.2%)	
**Overall travel times (this visit)**	**N (%)**		**Overall transportation costs**	**N (%)**		No drugs at the facility	12 (1.6%)	
≤15 min	377 (51.2%)		Less than US$1.00	696 (94.6%)		No one to accompany me	11 (1.5%)	
15 min - 29 min	237 (32.2%)		US$ 1.00 – US$4.00	36 (4.9%)		No female doctor/nurse	10 (1.4%)	
30 min - 44 min	45 (6.1%)		More than US$4.00	4 (0.5%)		If I’m out of town	8 (1.1%)	
45 min - 59 min	40 (5.4%)		Mean = US$ 0.37;			If I don't like the service	7 (1.0%)	
≥60 min	37 (5%)		Maximum = US$16.00			If the facility is closed	7 (1.0%)	
Mean = 30 minutes						If it is raining	1 (0.1%)	
Maximum = 300 minutes						If there's no one at home	1 (0.1%)	
**Elective termination of pregnancy**								
**Who carried out the procedure**	**N (%)**		**Where was the procedure done?**	**N (%)**		**What instrument was used?**	**N (%)**	
Doctor/nurse/midwife	33 (84.6%)		Facility	34 (87.2%)		A surgical instrument	29 (74.4%)	
Medicine store attendant/owner	2 (5.1%)		Home of abortionist	1 (2.6%)		A non-surgical instrument	3 (7.7%)	
Traditional medical practitioner	1 (2.6%)		Forest	-		Tablets/injections	6 (15.4%)	
Self medication/friend	3 (7.7%)		Other	4 (10.3%)		Don't know	1 (2.6%)	
**Any post abortion complications?**	**N (%)**		**Maternal age at the time of abortion**	**N (%)**				
Fever	14 (35.9%)		15 – 19 years	12 (32.4%)				
Prolonged vaginal bleed	8 (20.5%)		20 – 24 years	11 (29.7%)				
Others	7 (17.9%)		25 – 29 years	7 (18.9%)				
None	10 (25.6%)		30 – 34 years	2 (5.4%)				
			35 – 39 years	4 (10.8%)				
			40 – 44 years	1 (2.7%)				

The exchange rate used is $1 =  n156.22 (Nigerian naira), being the mean exchange rate for may 2011. Source: www.oanda.com.

N  =  sample size; DHS  =  Nigeria demographic and health survey 2008; TBA  =  traditional birth attendant; Ave  =  average.

* – **Means of transport today** applies to respondents only.

** – **Average cost** applies to both the respondent and her chaperone or who ever accompanied her (irrespective of how this person traveled).

### Facility Delivery

**Figure 1:**
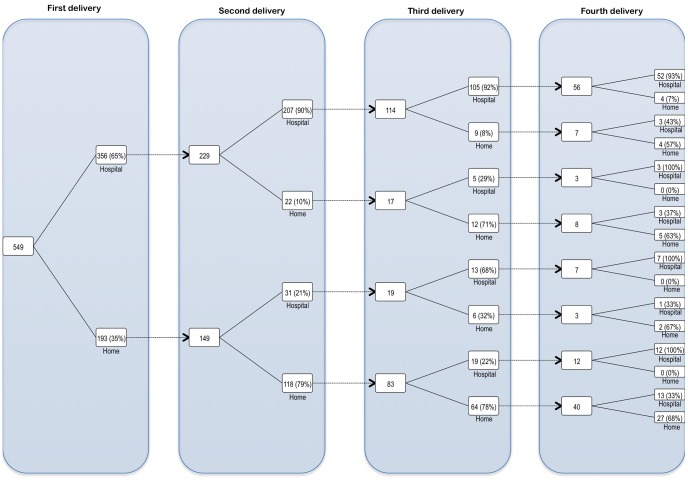
Reported deliveries by pregnancy order and delivery site. Three quarter (549) of our respondents reported having at least one delivery, while about half (378) reported at least two deliveries. From the latter group, deliveries were sorted by pregnancy order (up to the fourth delivery) and delivery site (with “**hospital**” representing all healthcare facilities, and “**home**” representing all other delivery sites e.g. the woman’s home, church, etc.) From this, we determined that the conditional probability of a facility delivery is 0.91 if it follows a previous facility delivery, and 0.24 if it follows a previous “**home**” delivery.

Two thirds of reported deliveries occurred in healthcare facilities (vs. 35% in 2008 DHS [16]), and of those that occurred at home, 10% were supervised by skilled attendants (vs. 4% in 2008 DHS [16]). To determine the probability of a facility delivery conditional on the previous delivery site, we limited the analysis to women who have had two or more deliveries. For every facility birth followed by a subsequent birth, 91% of those subsequent births occurred in a facility. Conversely, for every home birth followed by a subsequent birth, 24% of those subsequent births took place in a facility (see Figure 1). Using the same subset of respondents, we determined that if an adverse neonatal outcome (excluding low birth weight) occurred with a facility delivery, there is a significantly higher risk that the next delivery would occur at home. Conversely, if such an outcome followed a home delivery, the next delivery would most likely occur at home (see Table 3).

**Table 3:** Obstetric history of respondents alongside risk of various pregnancy and neonatal outcomes.

**Total number of reported pregnancies: 1,704**					
**Pregnancy outcome**	**N**	**Percent**	**Mode of delivery**	**N**	**Percent**
Live birth	1483	87.0%	Normal delivery	1162	94.5%
Still birth	68	4.0%	Forceps/vacuum delivery	19	1.2%
Spontaneous abortion	112	6.6%	Elective c/s	24	1.5%
Elective abortions	43	2.4%	Emergency c/s	42	2.5%
					
**Place of delivery**	N	Percent	**Neonatal outcomes**	N	Percent
Facility	983	63.2%	Low birth weight	77	5.0%
Home/Church/TBA House	572	36.8%	Neonatal death	63	4.1%
			Other complication	68	4.4%
			None	1328	86.5%
**Crude risk of a neonatal complication**	**RR (95% CI)**	**p-value**	**Crude risk of a still birth**	**RR (95% CI)**	**p-value**
By delivery site: facility (68/923) vs. home (63/535)	0.63 (0.45, 0.87)	0.005	By delivery site: facility (40/982) vs. home (28/572)	0.83 (0.52, 1.33)	0.445
By birth attendant: skilled (72/979) vs. unskilled(58/474)	0.60 (0.43, 0.83)	0.002	By birth attendant: skilled (41/1043) vs. unskilled(27/504)	0.73 (0.46, 1.18)	0.199
					
**Crude risk of neonatal death**	**RR (95% CI)**	**p-value**	**Consecutive birthing site (for** **deliveries between 2006–2011)**	**Prob. (95% CI)**	
By delivery site: facility (33/970) vs. home (30/565)	0.64 (0.40, 1.04)	0.069	Probability of a facility birth given apreceding facility birth (399/440)	0.91 (0.88, 0.93)	
By birth attendant: skilled (33/1031) vs. unskilled(29/499)	0.55 (0.34, 0.90)	0.015	Probability of a facility birth given apreceding home birth (75/307)	0.24 (0.19, 0.29)	
**Crude risk of switching delivery site after experiencing**			**Crude risk of switching birth attendant(s)** **for the next**		
**a neonatal complication**	**RR (95% CI)**	**p-value**	**delivery after experiencing a** **neonatal complication**	**RR (95% CI)**	**p-value**
Prior delivery **in** a health facility: 512 used,444 dropped	3.0 (1.58, 5.81)	0.001	A skilled BA oversaw prior delivery:545 used, 463 dropped	4.37 (2.20, 8.68)	<0.001
-Total complications = 38; subsequenthome birth = 9			-Total complications = 40; switched to anunskilled BA = 9		
-No complication = 474; subsequenthome birth = 37			-No complication = 505; switched to anunskilled BA = 26		
Prior delivery **outside** a health facility:370 used, 186 dropped	0.63 (0.31, 1.28)	0.178	An unskilled BA oversaw prior delivery:332 used, 162 dropped	0.90 (0.47, 1.74)	0.755
-Total complications = 44; subsequentfacility delivery = 7			-Total complications = 41; switched toa skilled BA = 8		
-No complication = 326; subsequentfacility delivery = 82			-No complication = 291; switched toa skilled BA = 63		
**BREASTFEEDING AND POSTPARTUM** **AMENORRHEA**					
**Newborn was exclusively breastfed**	n		**Duration of exclusive breast feeding**		
Yes	530 (35.5%)		Mean = 5.3 months		
No	961 (64.5%)		Median = 6 months		
					
**Duration of mixed feeding (from birth)**			**Duration of postpartum amenorrhea**		
Mean = 11.6 months			Mean = 9.3 months *(13.1 months in NDHS 2008)*		
Median = 12 months			Median = 8 Months *(11.5 months in NDHS 2008)*		

BA  =  Birth attendant; Prob.  =  Probability; CI  =  Confidence interval; NDHS  = 2008 Nigeria Demographic and Health Survey.

The average birth interval for all reported deliveries was **2.9** years, and it varied between **3.3** years for deliveries that occurred before year 2001, and **2.5** years for births that occurred from 2001.

## Discussion

This study provides the following insights. Firstly, most primary healthcare facilities in Nigeria are unable to adequately provide basic EmOC services or meet an increasing demand for obstetric care. To put this proper perspective, consider the following: of the 20,000 or so registered healthcare facilities in Nigeria (both public and private), about 80% are primary healthcare facilities, less than 1% are tertiary [3], [17] and referral facilities can seldom be found in rural areas (which harbor two thirds of the population) [18]. Even as the newly introduced Midwifery Service Scheme (MSS) has increased the availability of nurses and midwives in primary healthcare centers, service provision still remains low [19]. While most women will experience normal delivery, it is well documented that all women are at risk for pregnancy-related complications and resultant morbidity and mortality. It is for this very reason that primary healthcare centers accessible to all women are necessary but not sufficient to reduce maternal mortality. These facilities *also* need to be of high quality, with attendants that are capable of recognizing the need for expedient referral, and there *also* needs to be access to emergency transportation, high quality referral facilities capable of EmOC, and with capacity for C-section and blood transfusion in cases of extreme demise. Without attention to the type, quality, and distribution of facilities, Nigeria will be unable to reduce maternal mortality to the degree it aspires to. Additionally, these same concerns apply to neonatal care as most facilities lacked appropriate capacity here as well. There has been some concern that a disproportionate focus on tertiary facilities, at the expense of high quality primary facilities and accessible referral if needed for all women, has been politically motivated [20]. However, it is hoped that the newly signed National Health Bill would correct these inequalities [21].

Secondly, even though this was a selected sample of women who were able to get to a facility, many still indicated difficulty accessing care. Further, most of the women in this study reported living within 30 minutes of a healthcare facility; this supports the hypothesis that women living more than 30 minutes away from a facility are less likely to access facility-based care. Future studies are needed to determine the ‘threshold critical distance’ that serves as a barrier to accessing care – this will be important to consider when planning the optimal distribution of new facilities.. Our results also suggest that women who have a history of facility birth are likely to use a facility again. Therefore, efforts to increase use of facilities for childbirth should prioritize women not currently accessing facilities for childbirth. These findings have implications for the design of programs to reduce maternal and neonatal mortality, such as the *Abiye* project in Ondo state. The *Abiye* project connects pregnant women with skilled birth attendants via toll-free mobile phones, and includes efforts to improve the quality of select EmOC facilities that receive these women. As programs like this one are scaled up to cover a wider geographical area, it will be important to include specific strategies that target women who reside more than 30 minutes away from healthcare facilities; these are likely to require both improved transportation systems (emergency, public, etc.) and innovative ideas to enhance communication (e.g., toll-free phone).

Our results suggest that women who experienced adverse pregnancy outcomes in a facility may be less likely to seek facility-based obstetrical care in the future. While our study is able to describe this phenomenon, we are unable to ascertain the specific reasons women made this choice. For example, might women and their families assume that the adverse obstetrical outcome was secondary to poor quality of care? Are there new financial constraints as a result of the previous adverse event? Further studies are needed to identify the specific factors that contribute to this decision, as these factors will represent important areas of focus for programs trying to increase facility-based delivery.

This study has several limitations. First, our intention was not to make a comprehensive assessment of facility quality in a rigorous evaluation framework. This initial study was designed to inform assumptions that are necessary for our model-based analysis examining the costs and benefits associated with alternative strategies to reduce maternal mortality in in Nigeria. Studies that develop and validate criteria to assess facility quality will certainly be necessary to evaluate both ongoing and upcoming programs in Nigeria. The study design used also has limitations. For example results may have been influenced by misclassification (e.g. some questions may have been mistranslated as some respondents were interviewed in their local dialects), recall bias (e.g. malaria infestation may be responsible for most reported cases of postpartum fever), or survivor bias (e.g. the occurrence of reported maternal complications differed from published data [22]). The restriction of our study to a sample of women attending health facilities represents a select group of healthcare-seeking women; this hinders generalizability to all Nigerian women (e.g. our sample had greater proportions of younger, married, educated, employed and pregnant women than those in the 2008 DHS; see Table 2). That being said, within the limits study design and sample size, our sample may be reasonable representative of women who utilize healthcare facilities. In addition, we did not include a focus on any particular subgroup of women, for example women with HIV. Several critical questions related to pregnancy-related mortality and morbidity in HIV-infected women, factors influencing health seeking behavior and access to care - in the context of HIV prevalence rates above 5% - are deserving of focused study. Some of these are currently being addressed [23], [24], [25], [26], [27], [28] but additional work is needed.

In conclusion, reducing maternal deaths in Nigeria requires attention to increasing the number of facilities with EmOC capability, improving the quality of facilities, and both identifying and addressing the barriers facing Nigerian women in accessing these facilities. Despite limitations, our findings identify potentially important questions deserving of future study that could influence the design of new programs and policies.
